# Population-based survey of overweight and obesity and the associated factors in peri-urban and rural Eastern Uganda

**DOI:** 10.1186/s12889-015-2506-7

**Published:** 2015-11-24

**Authors:** Barbara Eva Kirunda, Lars Thore Fadnes, Henry Wamani, Jan Van den Broeck, Thorkild Tylleskär

**Affiliations:** Department of Epidemiology and Biostatistics, Makerere University College of Health Sciences, School of Public Health, P.O. Box 7072, Kampala, Uganda; Centre for International Health, University of Bergen, P.O. Box 7800, 5020 Bergen, Norway; Department of Clinical Dentistry, University of Bergen, P.O. Box 7800, 5020 Bergen, Norway; Department of Community Health and Behavioural Sciences, Makerere University College of Health Sciences, School of Public Health, P.O. Box 7072, Kampala, Uganda

**Keywords:** Overweight, Obesity, Associated factors, Peri-urban and rural Uganda

## Abstract

**Background:**

In sub-Saharan Africa (SSA), the rising prevalence of overweight, obesity and non-communicable diseases co-exists with the high burden of under-nutrition. The paucity of data on adulthood overweight and obesity, disaggregated by socio-demographic characteristics and in rural settings in SSA calls for research. We determined the prevalence of underweight, overweight/obesity and associated factors among adults in peri-urban and rural Uganda.

**Methods:**

A cross-sectional study of 1210 randomly selected adults aged ≥ 18 years was conducted in Iganga-Mayuge Health and Demographic Surveillance Site in eastern Uganda in 2013. Height, weight and socio-demographic variables were assessed. Overweight was defined as BMI = 25.0-29.99 kg/m^2^, obesity ≥ 30 kg/m^2^ and overweight/obesity ≥ 25 kg/m^2^. Logistic regression was used to identify factors associated with overweight/obesity.

**Results:**

Of the participants, 7 % were underweight (8.1 % of men; 5.9 % of women, *p* = 0.99); 17.8 % were overweight (12.4 % of men; 23.1 % of women, *p* < 0.001); and 7 % were obese (2.0 % of men; 12.7 % of women, *p* < 0.001). Overweight prevalence was 15.8 % and 23.8 % among rural and peri-urban adults, respectively (*p* < 0.001). Obesity prevalence was 3.9 % and 17.8 % among rural and peri-urban adults, respectively (*p* < 0.001). Factors associated with overweight/obesity were: being female, adjusted odds ratio (AOR) 4.3 (95 % confidence interval (PloS one 8:e75640, 20013) 3.2–5.9); peri-urban residence AOR 2.6 (1.9–3.6); being in age group 35–44, AOR 3.1 (1.8–5.3); 45–54 AOR 4.1 (2.3–7.3); 55–64 AOR 2.6 (1.4–5.0); ≥ 65 years AOR 3.1 (1.6–6.0); and having socio-economic status (SES) in the third AOR 2.8 (1.7–4.6), fourth 2.5 (1.5–4.2) and fifth 2.7 (1.6–4.4) quintile.

**Conclusions:**

Overweight/obesity was prevalent among adults. Overweight/obese was associated with being female, being aged 35 years and older, residing in a peri-urban area and having a higher SES. The time has come to develop interventions to prevent and control overweight/obesity.

**Electronic supplementary material:**

The online version of this article (doi:10.1186/s12889-015-2506-7) contains supplementary material, which is available to authorized users.

## Background

Worldwide, the prevalence of overweight and obesity are on the rise [1, 2] while the prevalence of under-nutrition has not significantly changed over the last decade [3]. While underweight prevalence is still high [4], overweight and obesity are now prevalent in low- and middle-income countries [4, 5], including those in Africa [1, 6], at a prevalence of 20–50 % [7–12] in urban areas and 7–30 % in rural areas [9–15].

In SSA, the rising prevalence of overweight and obesity co-exists with the under-nutrition epidemic [16, 17] and the increasing prevalence of non-communicable diseases (NCD) with an anticipated largest increase in NCD deaths of 27 % in Africa over the next decade [18]. Underweight, overweight and obesity are known risk factors for NCDs [19, 20]. Similarly, the Uganda Demographic and Health Surveys (UDHS) from 1995 to 2011 reported an increasing prevalence of overweight and obesity from 8 to 18.8 % while underweight prevalence stagnated at 10 – 12 % [21, 22].

However, evidence examining the influence of the individual, social and built environment on overweight/obesity is still patchy [23, 24] and limited to urban and suburban populations [24] in high-income countries which cannot be generalized to low- and middle-income countries [25] and this is even more true in rural settings.

The paucity of research on obesity and physical inactivity disaggregated by age, sex and residence [26] and the influence of the environment on overweight/obesity in Africa [25] calls for research in SSA [27] among peri-urban and rural populations in order to develop effective, culturally sensitive, context-specific and population-based interventions for the prevention of obesity and NCDs [25]. The objective of this study was to determine the prevalence of underweight, overweight and obesity and associated factors in a population-based sample of adults in peri-urban and rural Uganda.

## Methods

### Study design and setting

An observational cross-sectional study was conducted in 2013 among adults drawn from an active study cohort of 1 January 2005 to 30 September 2013 in the Iganga-Mayuge Health and Demographic Surveillance Site (IMHDSS) located in Iganga and Mayuge Districts in eastern Uganda, 120 km east of Kampala, the capital. The IMHDSS had an estimated population size of 80,000 people in 2013 of whom 51.2 % are females. The estimated number of households is 13,000 across an area of about 155 km^2^. The IMHDSS is composed of 65 villages with about 38 % of the population residing in peri-urban villages. Routine data collection is regularly carried out for births, deaths and their causes, marriages, in- and out-migration, education and socio-economic status. In addition to these routine surveillance activities, data are also collected for special studies.

### Study population and sampling

The study population comprised of men and non-pregnant women aged 18 years and above residing in the IMHDSS for at least 4 months, who had an individual identification IMHDSS number and were part of a household with an IMHDSS identification number. Adults who were ill, for whom physical activity was constrained or who were unable to communicate with the research team were excluded from participating in the study. Cluster sampling [28] was used to select participants from the active HDSS study cohort, with villages being the clusters. Probability proportionate to size sampling was used to select 40 villages, from which 30 households/participants were selected by simple random sampling using Stata data analysis and statistical software. From each village, an equal number of female and male respondents were randomly selected. In this study, a household was defined as a group of people who had been living and eating their meals together for at least 6 of the 12 months preceding the study. Study participants who were not found at their place of residence were replaced by individuals in neighbouring households who were matched by sex using the IMHDSS village lists.

### Data collection strategy

A field team of ten research assistants and one supervisor underwent a three-day standardized training on the study objectives, administration of the questionnaire and physical measurements of body weight and height. A semi-structured questionnaire was used to collect quantitative data on physical measurements of body weight and height and socio-demographic variables including sex, age, residence, marital status, religion, education level, occupation in the previous month, average monthly earnings and ownership of assets for purposes of assessing socio-economic status. Validated questions on socio-demographic variables were adopted from the IMHDSS [29] surveillance data collection tools and the Uganda Demographic and Health Survey [22]. The questionnaire was translated into the local language and pre-tested in the neighbouring district to check on the ease of comprehension of questions and anomalies were corrected. The field assistants worked in pairs composed of a nutrition assessor and an interviewer so as to optimize the quality of anthropometric measurements. The questionnaires from the field were checked daily for errors and missing data by the quality assurance officer.

### Outcome measure

For each study participant, anthropometric measurements of body height and weight were assessed using standard protocols, with subjects standing upright, not wearing shoes and wearing light weight clothes. Body height in centimetres (cm) was measured twice to the nearest 0.1 cm using Seca™ 213 portable stadiometers (Seca GmbH & Co. Kg., Hamburg, Germany). Body weight in kilograms (kg) was measured twice to the nearest 0.1 kg using calibrated Seca™ 876 digital weighing scale (Seca GmbH & Co. Kg., Hamburg, Germany). Final height and weight values were obtained as averages of the two measurements. The intraclass correlation coefficients (ICCs) for height and weight measurements within participants were 0.982 and 0.960 respectively, suggesting that intra-rater reliability was well above acceptable levels [30]. The outcome variable, body mass index was calculated and used as indicator of underweight (BMI <18.5 kg/m^2^), normal weight (BMI = 18.5 – 24.99 kg/m^2^), overweight (BMI = 25.0–29.99 kg/m^2^) and obesity (BMI ≥ 30 kg/m^2^) using the international classification of BMI [31–33]. For further analysis, overweight/obesity was defined as BMI ≥ 25 kg/m^2^ inclusive of the obese (BMI ≥ 30 kg/m^2^).

### Independent variables: socio-demographic factors

Age was recorded in complete years and significant past political or social local events were used as a proxy to estimate the ages of some respondents who did not know. Age in years was classified into 6 groups, namely 18 – 24, 25 – 34, 35 – 44, 45 – 54, 55 – 64 and ≥ 65 years. Marital status was classified as single, widowed/separated/divorced and cohabiting/married. Religion was classified as Catholic, Protestant, Other Christian, Moslem and Traditionalist. Residence was assessed using the HDSS peri-urban/rural classification which is based on population size, distance to Iganga town, access to amenities like piped water and mobility of the population. Education level was classified as none, lower primary, upper primary and secondary and above. Occupational activities in the previous month were classified as subsistence agriculture, commercial agriculture, casual labour, domestic work, student, trade and formal salaried employment. Average monthly earnings were assessed in Ugandan shillings (UGX) and classified as none, less than 60,000 (US$ 23), 60,000 – 100,000 (US$ 23 – 38) and more than 100,000 (US$ 38 (exchange rate September 2014: 1 US$ = UGX 2,620). Prior to creating the SES index, 27 items were checked for internal consistency using the Cronbach’s alpha measure. Fourteen items namely: 1) radio, 2) mobile phone, 3) bicycle, 4) motorcycle, 5) table, 6) machete, 7) axe, 8) hoe, 9) cattle, 10) goats, 11) poultry, 12) land ownership, 13) kerosene lantern, and 14) charcoal iron were identified (Cronbach’s alpha = 0.732) and used to create the SES index using principal component analysis. The factor scores of the first principal component were used to create 5 SES quintiles namely: first (poorest), second, third, fourth and fifth (least poor) [34].

### Statistical methods

Double data entry was done using EpiData version 3.1 software, cleaned and exported to IBM SPSS statistics 19 for analysis. Descriptive statistics were computed and expressed by socio-demographic characteristics and BMI. The prevalence estimates for underweight, normal weight, overweight and obesity were computed as percentages with the total sample size as the denominator. Crude odds ratios (COR) and their 95 % CI were computed to check for associations between categorical variables. All factors significantly associated with overweight/obesity (*p* < 0.10) in the bi-variable analysis such as sex, age, residence, marital status, occupational activities and socio-economic status, were included in the logistic regression model. In addition, an alpha level of 0.10 was used as a criterion for retaining a variable in the final logistic regression model. Logistic regression was used to identify factors associated with being overweight and obese using AOR at 95 % CI.

#### Ethical considerations

Ethical approval for the study was obtained from Makerere University School of Public Health Higher Degrees Research and Ethics Committee (IRB00011353) and the Uganda National Council for Science and Technology (HS1322). Permission to conduct the study in the HDSS was also sought from the Iganga-Mayuge HDSS steering committee and written informed consent was obtained from each participant.

We report following the Strengthening the Reporting of Observational Studies in Epidemiology (STROBE) Statement: Guidelines for reporting observational studies [35].

## Results

### Characteristics of participants

Of the 1210 participants, 50.1 % were women, 73 % were married/cohabiting, 55 % were Moslems, 12 % had no formal education and 60 % were involved in subsistence agriculture as the main source of livelihood, Table 1. The mean and median age of women in years were 42.5 ± 15.3 and 40.0 (Interquartile range (IQR) = 31.0–52.0). The mean and median age of men in years were 43.2 ± 16.6 and 42.0 (IQR = 30.0–54.0).

**Table 1:** Socio-demographic characteristics of study participants, *N* = 1210

Characteristics	Number *n*	Percent (%)
Sex		
Male	604	49.9
Female	606	50.1
Age groups		
18–24	167	13.8
25–34	247	20.4
35–44	297	24.5
45–54	222	18.3
55–64	137	11.3
≥65	140	11.6
Marital status		
Single	130	10.3
Widowed/separated/divorced	193	16.0
Cohabiting/Married	887	73.3
Religion		
Catholic	105	8.7
Protestant	364	30.1
Other Christian	74	6.1
Moslem	665	55.0
Traditionalist	2	0.2
Residence		
Peri-urban	298	24.6
Rural	912	75.4
Education level		
None	143	11.8
Lower primary	250	20.7
Upper primary	415	34.3
Secondary and above	402	33.2
Main occupation in the previous month		
Casual labour	85	7.0
Domestic work	73	6.0
Student	23	1.9
Subsistence agriculture	720	59.5
Trade	189	15.6
Commercial agriculture	69	5.7
Formal employment	51	4.2
Average monthly earnings (UGX^a^)		
None	113	9.3
<60000	458	37.9
60000-100000	344	28.4
>100000	295	24.4
SES^b^ quintiles		
First (poorest)	242	20.0
Second	244	20.2
Third	234	19.3
Fourth	263	21.7
Fifth (least poor)	227	18.8

^a^ UGX, Ugandan shillings, ^b^SES, socioeconomic status

### Prevalence of underweight, normal weight, overweight and obesity

The mean and median height, weight and BMI for women were 1.58 ± 0.07 m, 1.59 m (IQR = 1.54–1.63), 61.1 ± 13.1 kg, 58.8 kg (IQR = 51.6–67.8) and 24.4 ± 5.1 kg/m^2^, 22.1 kg/m^2^ (IQR = 20.9–26.4). The mean and median height, weight and BMI of men were 1.68 ± 0.06 m, 1.68 m (IQR = 1.64–1.72), 62.0 ± 9.8 kg, 60.3 kg (IQR = 55.9–67.2) and 21.9 ± 3.1 kg/m^2^, 21.3 kg/m^2^ (IQR = 19.9–23.0). The mean and median age and BMI were 42.8 ± 16.0 years, 41.0 (IQR = 30.0–53.0) and 23.2 ± 4.4 kg/m^2^, 22.1 kg/m^2^ (IQR = 20.3–25.0) kg/m^2^, Table 2.

**Table 2:** Age, height, weight and body mass index (BMI) by sex

	Age (years)	Height (m)	Weight (kg)	BMI, (kg/m^2^)
Women				
Mean	42.5	1.58	61.1	24.4
Median	40.0	1.59	58.8	22.1
SD^a^	15.3	0.07	13.1	5.1
IQR^b^	31.0–52.0	1.54–1.63	51.6–67.8	20.9–26.4
Men				
Mean	43.2	1.68	62.0	21.9
Median	42.0	1.68	60.3	21.3
SD^a^	16.6	0.06	9.8	3.1
IQR^b^	30.0–54.0	1.64–1.72	55.9–67.2	19.9–23.0
F test, *p*-value	4.93, 0.03	2.69, 0.10	36.44, 0.00	84.16, 0.00
Sexes combined				
Mean	42.8	1.63	61.6	23.2
Median	41.0	1.64	59.7	22.1
SD^a^	16.0	0.08	11.5	4.4
IQR^b^	30.0–53.0	1.57–1.69	54.2–67.4	20.3–25.0

^a^ SD, standard deviation, ^b^ IQR, interquartile range

Overall, 7 % of the participants were underweight, with no significant difference by sex (8.1 % of men vs. 5.9 % of women, *p* = 0.99). The prevalence of underweight was higher in rural areas at 8.1 % versus 4.0 % in the peri-urban areas, *p* = 0.26, Table 3. Among women of 55–64 years and ≥ 65 years, the prevalence of underweight was 14.1 % and 15.4 %, respectively; and among men, the prevalence of underweight was highest (17.3 %) in the oldest age group, 65 years and above, Additional file 1.

**Table 3:** Distribution of body mass index by sex, age and residence

Characteristics	Underweight %	Normal weight %	Overweight %	Obesity %	Chi-square value, *p*-value
Sex					
Male	8.1	77.5	12.4	2.0	85.3, < 0.001
Female	5.9	58.3	23.1	12.7	
*p*-value	0.99	Reference	<0.001	<0.001	
Age groups					
18–24	3.6	83.8	9.6	3.0	82.3, < 0.001
25–34	5.7	76.9	13.4	4.0	
35–44	5.1	62.6	23.6	8.8	
45–54	5.0	58.6	23.0	13.5	
55–64	11.7	65.0	15.3	8.0	
≥65	16.4	61.4	17.1	5.0	
Residence					
Peri-urban	4.0	54.4	23.8	17.8	82.0, < 0.001
Rural	8.1	72.3	15.8	3.9	
*p*-value	0.26	Reference	<0.001	<0.001	
Overall	7.0	67.9	17.8	7.4	

Eighteen percent of participants were overweight, with significant disparity by sex (12.4 % of men vs. 23.1 % of women, *p* < 0.001). Obesity was observed in 7 % of the respondents, with significant disparity by sex (2.0 % of men vs. 12.7 % of women, *p* < 0.001). The prevalence of overweight/obesity was 25.2 %. Overweight was more common in the peri-urban areas (23.8 %) compared to rural areas (15.8 %), *p* < 0.001, Table 3.

The prevalence of overweight was high in middle-aged adults, in age groups 35–44 years (29.1 % of women vs. 17.2 % of men) and 45–54 years (28.8 % of women vs. 17.1 % of men). In the younger age group, 25–34 years, the prevalence of overweight was high among women (21.8 %) compared to men (4.2 %). The prevalence of obesity was high (22.5 %) among women in the age group 45–54 years while the prevalence of obesity among men was highest (5.4 %) in the age group 55–64 years, Additional file 1.

**Fig. 1:**
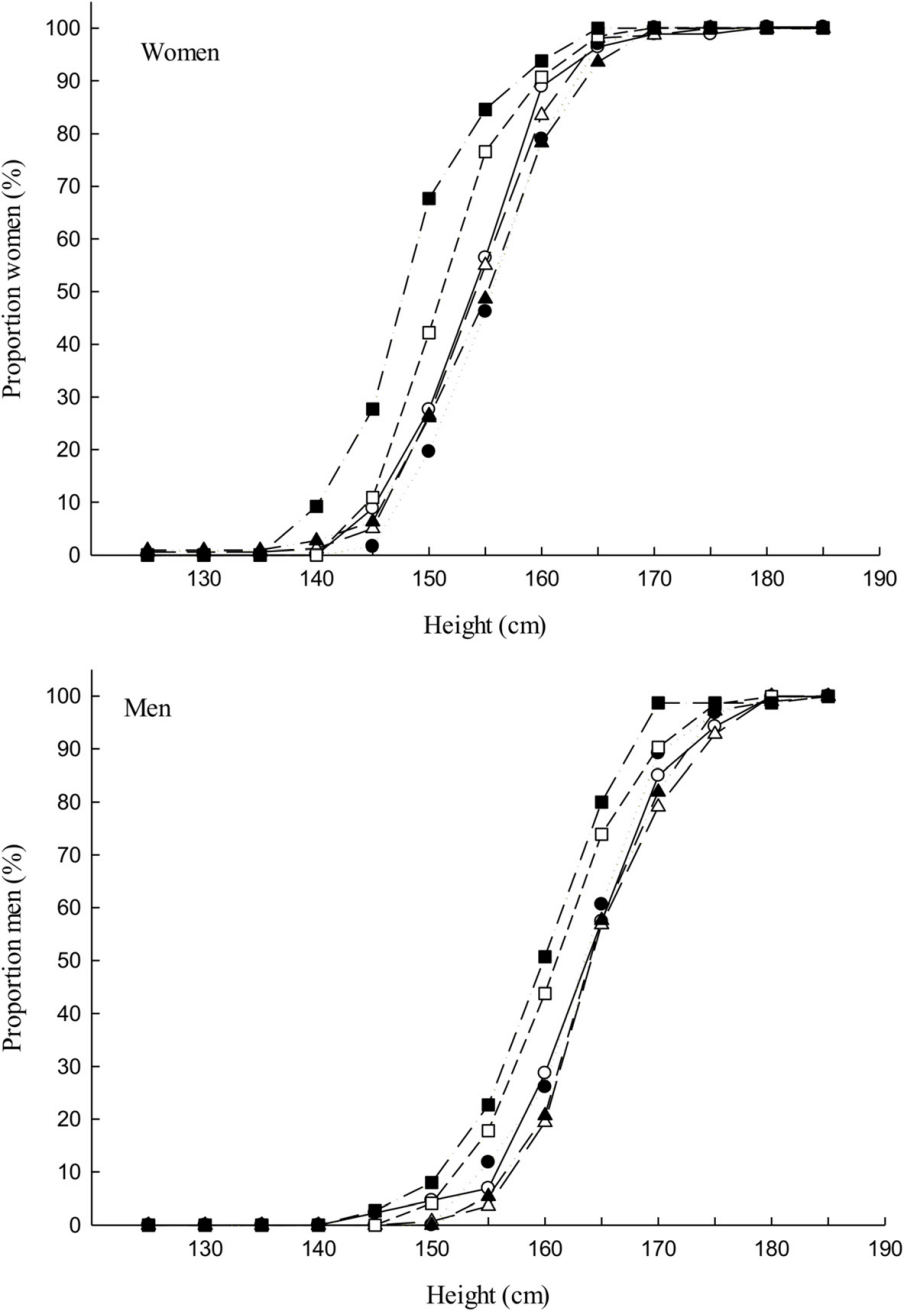
Distribution of body height (cm) by age group, *top*) women, *bottom*) men

**Fig. 2:**
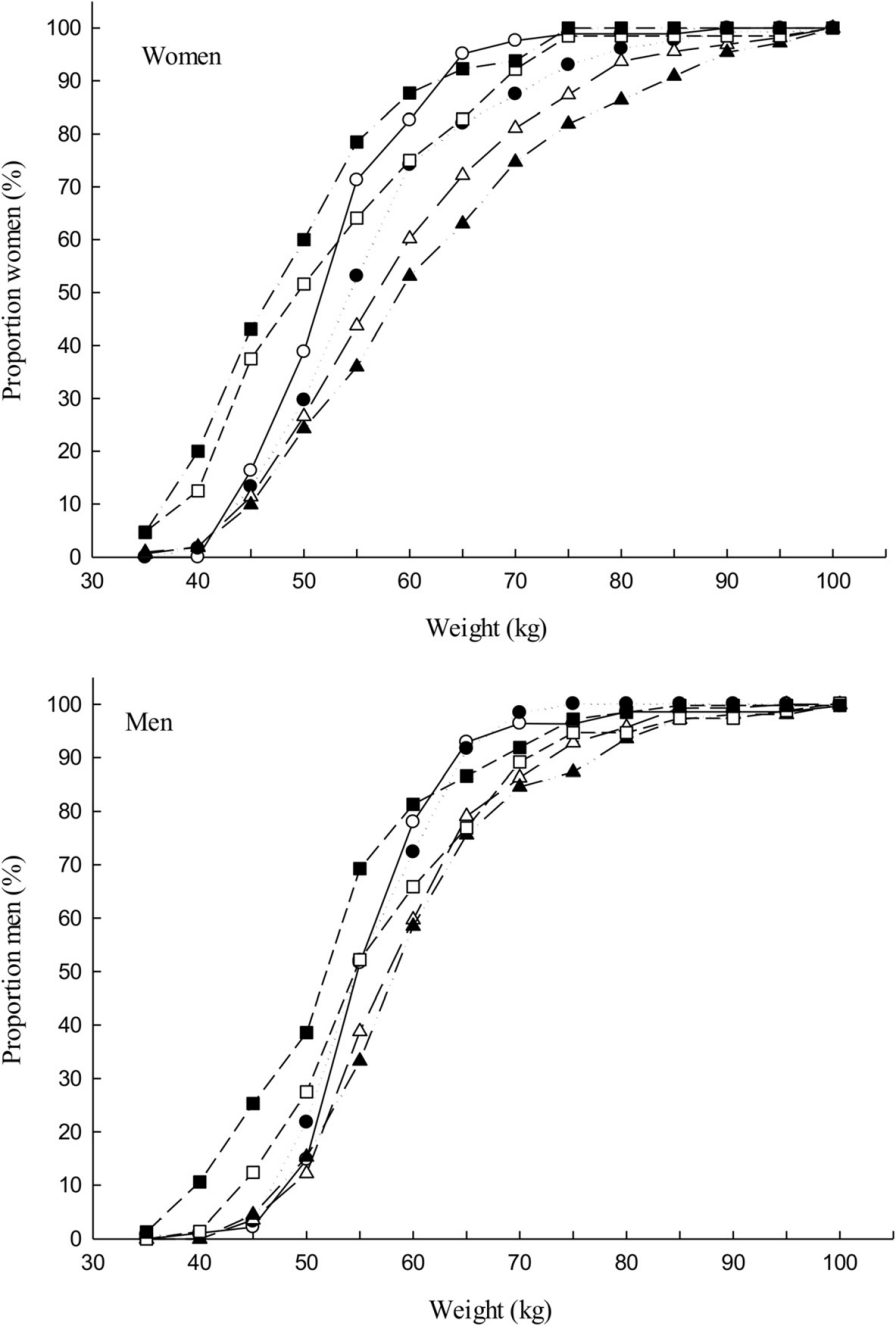
Distribution of body weight (kg) by age group, *top*) women, *bottom*) men

**Fig. 3:**
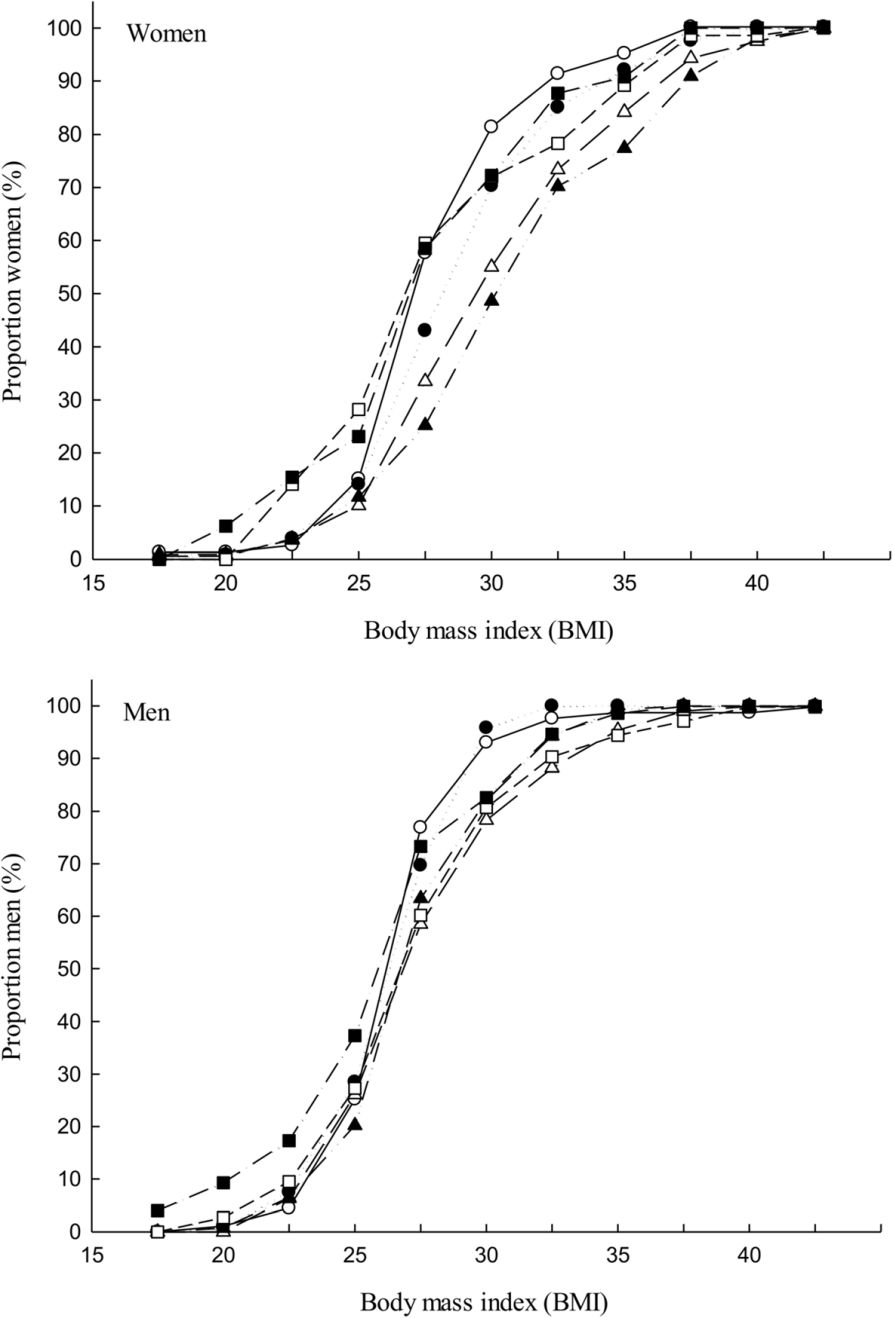
Distribution of BMI by age groups, *top*) women, *bottom*) men

Both men and women in the two oldest age groups (above 55 years) were shorter in height than those in the age groups below 55 years, Fig. 1. Among women, the age groups below 55 years were on average heavier than the age group ≥ 55 years while, for men, the middle age groups were heavier than other age groups, Fig. 2. Among the middle age groups (35–44 and 45–54), women had a much higher BMI compared to men, Fig. 3.

### Factors associated with overweight/obesity

Factors found to be associated with being overweight/obese were being female, age ≥ 35 years, peri-urban residence and higher SES. Females were 4 times more likely to be overweight/obese than males (AOR 4.3; 95 % CI 3.2–5.9). Participants who were aged 35–44 (AOR 3.1; 95 % CI 1.8–5.3), 45–54 (AOR 4.1; 95 % CI 2.3–7.3), 55–64 (AOR 2.6; 95 % CI 1.4–5.0) and ≥ 65 years (AOR 3.1; 95 % CI 1.6–6.0) were more likely to be overweight/obese than those aged 18–24 years. Peri-urban residents were 3 times more likely to be overweight/obese (AOR 2.6; 95 % CI 1.9–3.6) than rural residents. Participants who were in the third (AOR 2.8; 95 % CI 1.7–4.6), fourth (AOR 2.5; 95 % CI 1.5–4.2) and fifth SES quintiles (AOR 2.7: 95 % CI 1.6–4.4) were more likely to be overweight/obese than those in the first SES quintile, Table 4.

**Table 4:** Factors associated with being overweight or obese (body mass index, BMI ≥ 25 kg/m^2^), *N* = 1210

Characteristics	n	Overweight or obese %	Crude OR^a^ [95 % CI^b^]	Adj OR^a^ [95 % CI^b^]
Sex				
Male	604	14.4	1.0	1.0
Female	606	35.8	3.3 [2.5–4.4]	4.3 [3.2–5.9]
Age groups				
18–24	167	12.6	1.0	1.0
25–34	247	17.4	1.5 [0.8–2.6]	1.4 [0.8–2.5]
35–44	297	32.3	3.3 [2.0–5.6]	3.1 [1.8–5.3]
45–54	222	36.5	4.0 [2.3–6.8]	4.1 [2.3–7.3]
55–64	137	23.4	2.1 [1.2–3.9]	2.6 [1.4–5.0]
≥65	140	22.1	2.0 [1.2–3.6]	3.1 [1.6–6.0]
Residence				
Rural	912	19.7	1.0	1.0
Peri-urban	298	41.6	2.9 [2.2–3.8]	2.6 [1.9–3.6]
Marital status				
Single	130	10.8	1.0	
Widowed/separated/divorced	125	27.5	3.1 [1.7–5.9]	
Married/cohabiting	887	26.7	3.0 [1.7–5.4]	
Religion				
Christian/traditionalist	545	23.5	1.0	
Moslem	665	26.5	1.2 [0.9–1.5]	
Education level				
None	143	30.8	1.0	
Lower primary	250	24.8	0.7 [0.5–1.2]	
Upper primary	415	21.9	0.6 [0.4–1.0]	
Secondary and above	402	26.6	0.8 [0.5–1.2]	
Occupation in the previous month				
Casual labour	85	16.5	1.0	
Domestic work/student	96	32.3	2.4 [1.2–5.0]	
Subsistence agriculture	720	20.8	1.3 [0.7–2.4]	
Trade	189	40.7	3.5 [1.8–6.6]	
Commercial agriculture	69	20.3	2.8 [1.3–5.7]	
Formal employment	51	35.3	2.8 [1.2–6.2]	
Average monthly earnings (UGX^c^)				
None	113	22.1	1.0	
<60000	458	25.3	1.2 [0.7–2.0]	
60000-100000	344	25.3	1.2 [0.7–2.0]	
>100000	295	25.8	1.2 [0.7–2.0]	
SES^d^ quintiles				
First (poorest)	242	15.7	1.0	1.0
Second	244	18.9	1.3 [0.8–2.0]	1.5 [0.9–2.4]
Third	234	29.9	2.3 [1.5–3.6]	2.8 [1.7–4.6]
Fourth	263	28.1	2.1 [1.4–3.3]	2.5 [1.5–4.2]
Fifth (least poor)	227	33.5	2.7 [1.7–4.2]	2.7 [1.6–4.4]

^a^ OR odds ratio, ^b^ CI confidence interval, ^c^ UGX Ugandan shillings, ^d^ SES socioeconomic status

## Discussion

In this study of randomly selected adults in rural and peri-urban eastern Uganda, we found a relatively low burden of underweight and a high prevalence of overweight and obesity, particularly among middle aged women.

Our findings of a low prevalence of underweight was lower than findings in rural Uganda [14, 15], Kenya [9] and Congo-Brazzaville [36] probably as a result of variations in the age groups studied, definition and measurement of rural–urban divide. However, our findings were similar to those of African studies in Malawi [11], Cameroon [7], Ghana [37] and Nigeria [13, 38]. The higher prevalence of underweight among men (8.1 %) than women (5.9 %) is in consonance with findings elsewhere in Africa [7, 9, 10, 14, 15, 37, 39–41]; however, some studies in Africa have reported an insignificant inverse relationship [11, 13, 38].

This peri-urban and rural adult population already has a high burden of overweight (17.8 %) or obesity (7.4 %), the two combined affect 25.2 % of the population, comparable to contemporary findings in rural eastern Uganda (17.6 %) [15] but contrasting with rural south-western Uganda (11.3 %) [14], probably due to differences in the age composition of study populations. Our findings are consistent with findings in Africa of an overall overweight/obesity prevalence of 21.9 % in Malawi [11], 29.2 % in Nigeria [13], 20.1 % of the normotensive and 22.2 % of the hypertensive adult populations in Nigeria [42], and 27.1 % of rural residents in Ghana [37]. The overall prevalence estimate of overweight/obesity is less than the estimates in a few studies in Africa, such as 37.1 % in Ghana [37], and 31.6 % in Nigeria [38], probably as a result of differences in the definition and measurement of the rural–urban divide.

Being female was the most significant factor associated with being overweight/obese. This is in consonance with findings in Uganda [15], Mozambique [10], Malawi [11] and Zambia [43]. A higher prevalence of overweight, obesity and overweight/obesity among women than men are consistent with findings in Uganda [14, 15]. These findings are consistent with findings reported elsewhere in Africa for instance in Nigeria [38, 44], Kenya [9], Mozambique [10], Malawi [11], Botswana [40], Ghana [37], Zambia [43], Tanzania [45], Algeria [39], South Africa [41, 46] and Cameroon [7]. However, they differ from the findings of one study in Nigeria [13] and studies in high income countries where men have a higher prevalence of overweight/obesity than women [47–49]. These differences can probably be explained by behavioural factors given that both men and women are exposed to the genetic, physical and social environment [12, 50]. In addition, studies in Africa have reported a social norm of acceptability, or even preference for overweight and obesity particularly among women and it is perceived to be associated with affluence [51–54].

Age was another factor found to be associated with overweight/obesity and has been found in other studies in Africa [10, 11, 15, 40]. It has also been observed that the prevalence of overweight/obesity was highest in the age group 45–54 years and this has been confirmed elsewhere in Africa [10, 11, 15]. Similarly mean BMI was highest in the middle age group, more evidently among the urban than rural residents in Kenya [9] and in Mozambique [10].

Our study affirms that peri-urban residence was another significant factor, similar to findings in Africa [15, 40] but contrasting with findings in urban Zambia [43]. It was also observed that the prevalence of overweight/obesity of 41.6 % among peri-urban residents is comparable to 35.7 % reported in peri-urban eastern Uganda [15]. Our study estimates of overweight and obesity of 23.8 % and 17.8 % among peri-urban residents, respectively, are comparable to 18.5 % and 13.1 % of overweight and obesity respectively in Nigeria [38] and also comparable to findings in Zambia [43], Malawi [11], Ghana [37], Mozambique [10], Tanzania and Namibia [55]. Our prevalence estimate is lower than findings in South Africa [56, 57]. The prevalence of obesity among rural residents in our study is comparable to findings in rural Nigeria [44], Malawi [11] and Kenya [9]. A higher prevalence of overweight and obesity among peri-urban residents compared to rural residents could be attributed to rural residents being more actively involved in labour intensive subsistence farming [12] than peri-urban residents whose occupations may encourage sedentariness which is more common in urban areas. However, most studies in Africa have reported a higher prevalence of overweight and obesity in urban areas than to rural areas [9–12, 37, 39, 40, 58–61]. The data is often limited to an urban–rural dichotomy instead of presenting the whole spectrum and therefore, there is paucity on data on overweight and obesity among peri-urban populations in Africa.

An increase in the prevalence and likelihood of being overweight/obese was observed with increasing SES in our study, indicating that this population could be vulnerable to co-morbidities associated with being overweight or obese. These findings could be explained by the availability of affordable, high energy-dense foods due to urban sprawl, and reduced physical activity resulting into a sedentary lifestyle as observed in a study in Kenya where women who were most sedentary were in the highest income group [62] with the ability to purchase energy-dense foods [63]. These findings have been confirmed in studies in rural Uganda [15], elsewhere in Africa [45, 62] and other low- and middle income countries [64], but are in contrast to findings in the high income countries, where the low-income earners are most likely to be overweight/obese. Due to differences in methods of measurement, analysis and categorisation of SES, cross-study comparisons were difficult and limited.

### Strengths and limitations

The main strengths of our study include a large population based, representative study sample which allows comparisons, and a wide age group of 18–92 years, which included younger and older adults, who are often left out of surveys. A small number of trained measurers conducting repeated anthropometric measurements provided fairly accurate and consistent estimates. The SES index was based on relevant specific household items after checking the reliability of each of the items using item analysis. The international classification of BMI recommended by the WHO was used for categorisation.

The study had some methodological limitations. It was cross-sectional in nature and therefore we cannot infer causality. However, the motivation was to describe the distribution of BMI and provide cues to potential associations that can be further explored using robust study designs. Despite the inherent limitations of BMI as a measure of weight status, it remains the most widely used measure for assessing weight status in populations. We acknowledge the relevance of assessing proximate factors for overweight/obesity such as physical activity and dietary intake; however, this was beyond the scope of this study. Given that the study was undertaken in the IMHDSS, where there is continuous data collection of demographic characteristics and special studies, during the time of the study, there were no interventional studies on NCDs. Data on human immunodeficiency virus (HIV) was not collected and yet it could have confounded the anthropometric measurements given that wasting and lipodystrophy are characteristic presentations [65]. However, HIV prevalence in the East Central Region where the IMHDSS is located is relatively low at 5.8 % [66], which would not confound the overall picture. Data on the physical and social environment were not collected and yet there are indications that the environment influences lifestyle behaviours such as physical activity and diet, which have an impact on the prevalence of overweight and obesity.

## Conclusions

In the predominantly rural adult population, the prevalence of overweight and overweight/obesity are already high, particularly among women, middle aged adults, peri-urban residents and adults in the higher quintiles of SES. Overweight/obesity appears to occur in significant proportions among young adults, particularly the women. The time has come to try to prevent and control overweight/obesity. Further inquiry is required to understand the perceptions of weight, diet and physical activity in the general population, and describe dietary diversity and physical activity, as well as the influence of the rural physical and social environment on diet, physical activity and BMI so as to guide the design and implementation of appropriate strategies for the prevention and control of overweight and obesity in similar populations.

## Additional file

Additional file 1:
**Distribution of body mass index by age, sex and sexes combined.** Description of data: The table provides details on the distribution of different groups of body mass index stratified by sex and age groups. (XLSX 11 kb)
